# *Lactobacillus acidophilus*-Fermented Germinated Brown Rice Suppresses Preneoplastic Lesions of the Colon in Rats

**DOI:** 10.3390/nu11112718

**Published:** 2019-11-09

**Authors:** Sing-Chung Li, Han-Pei Lin, Jung-Su Chang, Chun-Kuang Shih

**Affiliations:** 1 School of Nutrition and Health Sciences, College of Nutrition, Taipei Medical University, Taipei 11031, Taiwan; sinchung@tmu.edu.tw (S.-C.L.); pefaule123@gmail.com (H.-P.L.); susanchang@tmu.edu.tw (J.-S.C.); 2 Graduate Institute of Metabolism and Obesity Sciences, College of Nutrition, Taipei Medical University, Taipei 11031, Taiwan; 3 School of Food Safety, College of Nutrition, Taipei Medical University, Taipei 11031, Taiwan; 4 Master Program in Food Safety, College of Nutrition, Taipei Medical University, Taipei 11031, Taiwan

**Keywords:** germinated brown rice, fermentation, *Lactobacillus acidophilus*, colorectal cancer, apoptosis, inflammation

## Abstract

Colorectal cancer (CRC) is a cancer associated with chronic inflammation. Whole grains and probiotics play a protective role against CRC. Fermented grains are receiving increased attention due to their anti-inflammatory and anti-cancer activities. Our previous study found that a combination of germinated brown rice (GBR) with probiotics suppressed colorectal carcinogenesis in rats. However, the cancer-preventive effect of probiotic-fermented GBR has not been reported. This study investigated the preventive effect and possible mechanism of GBR fermented by *Lactobacillus acidophilus* (FGBR) on colorectal carcinogenesis in rats induced by 1,2-dimethylhydrazine (DMH) and dextran sulfate sodium (DSS). DMH/DSS treatment induced preneoplastic aberrant crypt foci (ACF), elevated serum levels of tumor necrosis factor (TNF)-α, interleukin (IL)-6 and IL-1β, as well as decreased pro-apoptotic Bax expression. GBR and FGBR reduced the primary ACF number and decreased TNF-α, IL-6 and IL-1β levels. GBR and FGBR at the 2.5% level increased pro-apoptotic cleaved caspase-3 and decreased anti-apoptotic B-cell lymphoma 2 (Bcl-2) expressions. FGBR at the 2.5% level further reduced the number of sialomucin-producing ACF (SIM-ACF) and increased Bax expression. These results suggest that FGBR may inhibit preneoplastic lesions of the colon via activating the apoptotic pathway. This fermented rice product may have the potential to be developed as a novel dietary supplement for CRC chemoprevention.

## 1. Introduction

Colorectal cancer (CRC) ranks third in terms of incidence and second in terms of mortality among all cancers in the world [1]. The highest CRC incidence rates are in parts of Europe, Australia/New Zealand, Northern America and Eastern Asia [1]. The rise in CRC incidence is related to dietary patterns, obesity and some lifestyle factors [1]. Chronic inflammation is considered among the hallmarks of cancer [2]. CRC is among the best examples of cancer closely associated with chronic inflammation, which can be present from the initiation of cancer [2]. Several studies show that key inflammatory signaling pathways, such as nuclear factor kappa-light-chain-enhancer of activated B cells (NF-κB), mitogen-activated protein kinase (MAPK), protein kinase B/phosphoinositide 3-kinase (Akt/PI3K) and peroxisome proliferator-activated receptor γ (PPARγ), are targets of probiotics or their products [3]. Metabolites of probiotics exhibit good anti-inflammatory and antioxidative properties in intestinal epithelial cells (IECs) and immune cells, and thus they can be used as an adjuvant in anti-inflammatory therapy [4].

Probiotic foods are fermented products containing sufficient amounts of certain live microorganisms that beneficially modify the intestinal microbiota of the host [5]. The most common probiotic foods are fermented dairy products such as yogurt [6]. Due to the vegan diet, traditional and economic causes, as well as some adverse effects such as lactose intolerance and allergy, the use of dairy products is limited in many countries, thus resulting in the requirement of nondairy probiotic foods [7]. Cereals are among the most promising alternatives to milk due to their ability to support the growth of probiotics and their protective bile-resistant effect [5]. The choice of different cereals and germination procedures can provide tailored substrates for the growth of probiotics [8]. Cereal fermentation is the most simple and economical method to improve the nutritional value, sensory properties and functional qualities [9,10,11]. A lot of plant polyphenols are biologically unavailable in the gastrointestinal tract, and fermentation may improve their bioavailability and bioactivity [12]. Fermented foods are receiving increased attention in research due to their proven safety and potential ability to prevent and treat several chronic diseases [13].

Rice contains many essential nutrients to support the growth of probiotics and can directly be used as the substrate for microbial fermentation [5,14]. For example, brown rice and rice bran are suitable substrates for the culture of *Lactobacillus plantarum* [5]. In addition, both brown rice and germinated brown rice (GBR) can be used as a supplement for *Lactobacillus*-mediated fermentation [14]. However, a number of studies indicated that the growth of *Bifidobacteria* spp. in cereal substrates is difficult unless a growth promoter (milk or yeast extract) is added [9]. These studies suggest that probiotics from genus *Lactobacillus* may be important starters for rice fermentation in food industry. The process of fermenting rice with bacteria or fungi can beneficially alter the bioactivity [12]. Some fermented rice products have been demonstrated to possess an anti-cancer effect. For example, brown rice and rice bran fermented by *Aspergillus oryzae* (FBRA) significantly suppressed colorectal and inflammation-related carcinogenesis in different animal models [15,16,17]. The water extract of FBRA also showed an induction of apoptosis in human colon cancer cells [18]. In addition, the ethyl acetate extract of *Phellinus linteus* grown on GBR (PBR) induced apoptotic cell death in human colon cancer cells [19]. The ethanol extract of PBR increased the sensitivity of cetuximab, a monoclonal antibody against KRAS-mutated colon cancer [20]. Either ethanol or the ethyl acetate extract of *Antrodia camphorata* grown on GBR (CBR) also showed an antiproliferative effect in human colon cancer cells [21].

Although there have been some studies examining the inhibitory effect of fermented products of brown rice, rice bran and GBR on colorectal carcinogenesis, most of them use certain fungi to mediate the fermentation process. In addition, the majority of these studies focused on fermented GBR products are *in vitro* studies, and thus the *in vivo* anti-cancer effect is not clear. Our previous study has found that a synbiotic combination of GBR with *Lactobacillus acidophilus* and/or *Bifidobacterium animalis* subsp. *lactis* inhibits colorectal carcinogenesis in rats [22]. However, the anti-cancer effect of GBR fermented by probiotics is still unknown. The present study was designed to investigate the effect of GBR fermented by *L. acidophilus* (FGBR) on colorectal carcinogenesis using a chemically induced animal model to elucidate the possible mechanism.

## 2. Materials and Methods

### 2.1. Preparation of GBR and FGBR

GBR is a product of AsiaRice Biotech, Inc. (Taipei, Taiwan). For GBR preparation, brown rice (*Oryza sativa*, Taikeng No. 9) was soaked in water at 37 °C for 22 h. Raw GBR was mixed with water (1:0.7, *w*/*v*), sterilized at 121 °C for 15 min, frozen-dried and screened through a 40 mesh sieve to obtain GBR powder. For FGBR preparation, GBR was mixed with water (1:19, *w*/*v*), sterilized at 121 °C for 15 min, added with 10% (*w*/*v*) of *L. acidophilus* (Chr. Hansen Holding A/S, Hoersholm, Denmark) at 37 °C for 24 h, and finally frozen-dried to obtain FGBR powder.

### 2.2. Animals and Diets

The protocol of the animal study was approved (LAC-2013-0302) by the Institutional Animal Care and Use Committee (IACUC) of Taipei Medical University. Sixty-six male F344 rats (3 or 5 weeks old) were purchased from the National Laboratory Animal Center (Taipei, Taiwan). Rats were housed in plastic cages in a room under a controlled temperature of 21 ± 2 °C and 40–60% relative humidity, with a 12 h light/dark cycle. Rats had free access to diet and water. After acclimatization, rats were divided into six groups and fed the purified diet for growing laboratory rodents established by American Institute of Nutrition (AIN-93G diet) in blank (group B) and control (group D) or fed the modified AIN-93G diet containing 10% GBR (group G), 2.5% FGBR (low dose, group LF), 5% FGBR (medium dose, group MF) and 10% FGBR (high dose, group HF). The above doses were percentages in feed based on dry matter. Rats were fed the experimental diets 1 week prior to carcinogen treatment. All rats except for those in group B were intraperitoneally injected with 1,2-dimethylhydrazine (DMH, Sigma-Aldrich Inc., St. Louis, MO, USA) at 40 mg/kg body weight three times in a week, and then fed 2% dextran sulfate sodium (DSS, Sigma-Aldrich Inc., St. Louis, MO, USA) in drinking water after the third DMH injection for 1 week. Body weight and food intake were recorded weekly. After 10 weeks of experimental period, rats were anaesthetized with isoflurane and blood (8 mL/rat) was collected from the abdominal aorta. Colons were also collected for evaluation.

### 2.3. Analysis of Aberrant Crypt Foci (ACF)

Colonic ACF were analyzed using the method established by Bird (1987) [23]. Briefly, the colon was removed, cut along the longitudinal axis and flushed with saline solution. Each colon was cut into three (proximal, middle and distal) sections with equal length and fixed flat between filter papers in 10% buffered formalin (Sigma-Aldrich Inc., St. Louis, MO, USA) for at least 24 h. The fixed colon sections were stained with 0.2% methylene blue solution (Showa Chemicals Co., Tokyo, Japan) for 3 min, and then ACF were observed using a light microscope (Nikon Corp., Tokyo, Japan) and their location were recorded. The area of each colon section was calculated by NIS-Elements microscope imaging software (Nikon Corp., Tokyo, Japan). The numbers of ACF and aberrant crypt (AC) in each focus were presented as numbers/cm^2^.

### 2.4. Analysis of Mucin-Producing ACF and Mucin-Depleted Foci (MDF)

Colonic mucin-producing ACF and MDF were analyzed using the high-iron diamine alcian blue (HIDAB) staining method and identified by the criteria established by Jenab et al. (2001) and Caderni et al. (2003) [24,25]. Briefly, the methylene blue-stained colon was faded with 70% ethanol (Taiwan Tobacco and Liquor Co., Taipei, Taiwan), immersed in high-iron diamine solution (Sigma Chemical Co., St. Louis, MO, USA) protected from light at room temperature for 50 min, rinsed in saline solution, stained with 1% alcian blue (Sigma Chemical Co., St. Louis, MO, USA) dissolved in 3% acetic acid (Shimakyu’s Pure Chemicals, Osaka, Japan) for 30 min, rinsed in 80% ethanol (Taiwan Tobacco and Liquor Co., Taipei, Taiwan) followed by saline solution, and finally observed using a light microscope (Nikon Corp., Tokyo, Japan). Brown- and blue-staining by HIDAB solution represented sulfomucin (SUM) and sialomucin (SIM) secretion, respectively. SUM-producing ACF (SUM-ACF) and SIM-producing ACF (SIM-ACF) were defined as ACF with over 85% SUM- and SIM-stained area, respectively. ACF stained with a smaller percentage of these two mucins were defined as mixed-type ACF (MIX-ACF). Foci without mucin production were defined as MDF. The area of each colon section was calculated by NIS-Elements microscope imaging software (Nikon Corp., Tokyo, Japan). The numbers of mucin-producing ACF and MDF were presented as numbers/cm^2^.

### 2.5. Analysis of Cytokines

The serum was separated by centrifugation at 3000× *g* at 4 °C for 15 min and stored at −80 °C until analysis. The levels of tumor necrosis factor (TNF)-α, interleukin (IL)-6 and IL-1β were detected by enzyme-linked immunosorbent assay (ELISA, PeproTech, Jersey City, NJ, USA).

### 2.6. Analysis of Protein Expression

Half of each colon section was examined for ACF, while the other half was analyzed for protein expression. Colonic tissues were homogenized in radioimmunoprecipitation assay (RIPA) lysis buffer (pH 7.5, 150 mM sodium chloride, 1% NP-40, 0.5% sodium deoxycholate, 0.1% sodium dodecylsulfate, 50 mM Tris, and 10% protease inhibitor mix). The homogenates were centrifuged at 10,000× *g* at 4 °C for 15 min, and then supernatants were separated and kept at −80 °C until analysis. The protein concentration was determined using the Bio-Rad Bradford assay (Bio-Rad, Hercules, CA, USA). Protein samples (20 μg) were separated by 4–12% sodium dodecylsulfate polyacrylamide gel electrophoresis and transferred to a polyvinylidene difluoride (PVDF) membrane. The membranes were incubated with rabbit primary polyclonal antibodies against Bax (1:1000 dilution, Cell Signaling Technology, Inc., Beverly, MA, USA), B-cell lymphoma 2 (Bcl-2, 1:1000 dilution, Cell Signaling Technology, Inc., Beverly, MA, USA) and caspase-3 (1:1000 dilution, GeneTex, Inc., San Antonio, TX, USA) at 4 °C overnight. After washing three times in Tris-buffered saline containing 1% Tween 20 (TBST), the membranes were incubated with an anti-rabbit IgG conjugated with alkaline phosphatase at room temperature for 1 h, washed and incubated with 5-bromo-4-chloro-3-indolyl-phosphate/nitroblue tetrazolium (BCIP/NBT) substrate solution (Sigma-Aldrich Inc., St. Louis, MO, USA) in the dark. The signals were quantitated with the Bio-Rad Quantity One software (Bio-Rad Laboratories, Hercules, CA, USA).

### 2.7. Statistical Analysis

Data are expressed as the mean ± standard deviation (SD) or percentage. The difference between groups was assessed by one-way analysis of variance (ANOVA) followed by Duncan’s multiple range test using SAS 9.4 software (SAS Institute, Cary, NC, USA). The statistical analysis of incidence was performed using Chi-square test. Correlation analysis was carried out using Spearman correlation. *p* < 0.05 was considered statistically significant.

## 3. Results

### 3.1. Effects of GBR and FGBR on Body Weight and Food Intake in Rats

There were no differences in weight gain and food efficiency among groups, indicating that both GBR and FGBR did not affect the growth of rats.

### 3.2. Effects of GBR and FGBR on Colonic ACF in Rats

**Figure 1:**
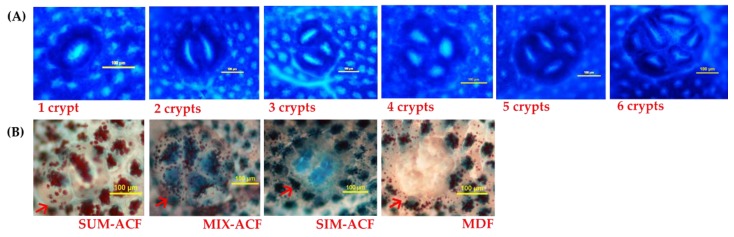
Preneoplastic lesions observed in the colon of F344 rats induced by 1,2-dimethylhydrazine/dextran sulfate sodium (DMH/DSS). (**A**) Methylene blue-stained aberrant crypt foci (ACF) with different numbers of crypts. (**B**) High-iron diamine alcian blue (HIDAB)-stained ACF with different types of mucin and mucin-depleted foci (MDF).

ACF incidence was 100% in all DMH/DSS-treated groups (American Institute of Nutrition (AIN)-93G diet, control (D); AIN-93G containing 10% GBR (G); AIN-93G containing a low dose (2.5%) of FGBR (LF); AIN-93G containing a medium dose (5%) of FGBR (MF); AIN-93G containing a high dose (10%) of FGBR (HF), whereas there was no ACF in group B (data not shown). The representative methylene blue-stained ACF containing different numbers of crypts are shown in Figure 1A. As shown in Table 1, rats fed GBR (group G) and all doses of FGBR (groups LF, MF and HF) had significantly lower numbers of ACF containing one AC, the primary ACF, than the control (group D, *p* < 0.05). Rats fed 2.5% FGBR (group LF) had significantly lower numbers of ACF containing two and three ACs as well as total ACF and total AC than the control (group D, *p* < 0.05).

### 3.3. Effects of GBR and FGBR on Mucin Secretion and MDF in the Colon of Rats

The representative HIDAB-stained ACF-producing mucins are shown in Figure 1B. As shown in Table 2, rats fed 5% and 10% FGBR (groups MF and HF) had significantly higher numbers of MIX-ACF (*p* < 0.05), while rats fed 2.5% FGBR (group LF) had significantly lower numbers of SIM-ACF (*p* < 0.05) than the control (group D). There was no MDF in rats fed 2.5% FGBR (group LF).

### 3.4. Effects of GBR and FGBR on Serum Pro-Inflammatory Cytokines in Rats

**Figure 2:**
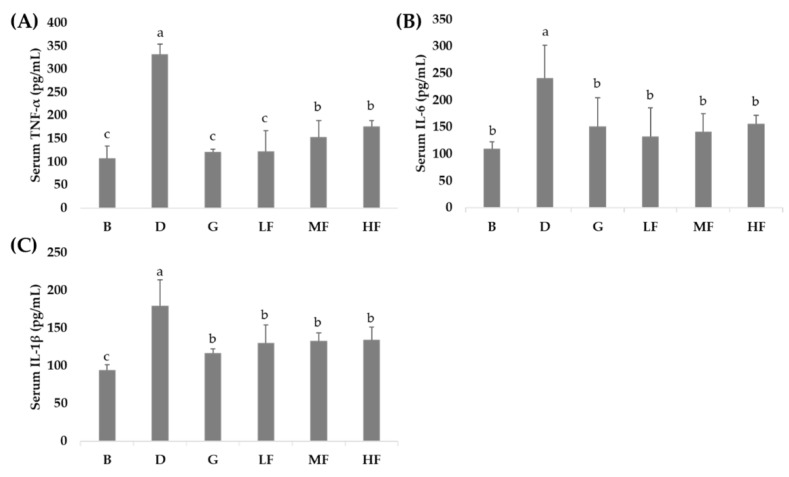
Effects of GBR and FGBR on the serum levels of tumor necrosis factor (TNF)-α (**A**), interleukin (IL)-6 (**B**) and IL-1β (**C**) in F344 rats. The bars represent the mean ±SD (*n* = 3). Values with the same letter are not significantly different from one another as determined by Duncan’s multiple range test, *p* < 0.05. All rats except those in group B were administered with DMH/DSS. B (blank) and D (control): AIN-93G diet; G: AIN-93G containing 10% GBR; LF: AIN-93G containing a low dose (2.5%) of FGBR; MF: AIN-93G containing a medium dose (5%) of FGBR; HF: AIN-93G containing a high dose (10%) of FGBR.

As shown in Figure 2, the serum levels of TNF-α, IL-6 and IL-1β were significantly higher (*p* < 0.05) in control (group D) compared to blank (group B). Rats fed GBR (group G) and all doses of FGBR (groups LF, MF and HF) had significantly lower serum levels of TNF-α, IL-6 and IL-1β (*p* < 0.05) than the control (group D).

### 3.5. Effects of GBR and FGBR on the Expression of Apoptosis-Related Proteins in the Colon of Rats

**Figure 3:**
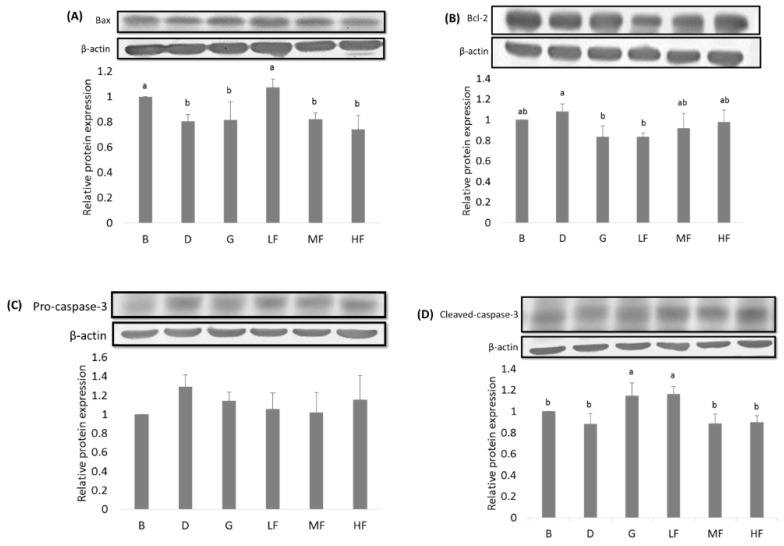
Effects of GBR and FGBR on the colonic expressions of (**A**) Bax (20 KDa), (**B**) Bcl-2 (26 KDa), (**C**) pro-caspase-3 (32 KDa) and (**D**) cleaved caspase-3 (16 KDa) in F344 rats. The bars represent the mean ±SD (*n* = 3). Values with the same letter are not significantly different from one another as determined by Duncan’s multiple range test, *p* < 0.05. All rats except those in group B were administered with DMH/DSS. B (blank) and D (control): AIN-93G diet; G: AIN-93G containing 10% GBR; LF: AIN-93G containing a low dose (2.5%) of FGBR; MF: AIN-93G containing a medium dose (5%) of FGBR; HF: AIN-93G containing a high dose (10%) of FGBR.

As shown in Figure 3, the expression of pro-apoptotic Bax was significantly lower (*p* < 0.05) in control (group D) compared to blank (group B). Rats fed 2.5% FGBR (group LF) had significantly higher Bax expression (*p* < 0.05) than the control (group D). Rats fed GBR (group G) and 2.5% FGBR (group LF) had significantly lower expression of anti-apoptotic Bcl-2 (*p* < 0.05) and higher expression of pro-apoptotic cleaved caspase-3 (*p* < 0.05) compared to the control (group D). There was no difference in pro-caspase-3 expression among groups.

## 4. Discussion

This is the first study showing a preventive effect of GBR fermented by *L. acidophilus* on colorectal carcinogenesis in rats. In this study, we used ACF as a morphological marker and both SIM-ACF and MDF as dysplastic markers during colorectal carcinogenesis, as mentioned by a previous study [26]. We also measured the serum levels of pro-inflammatory cytokines and the colonic expressions of apoptosis-related proteins to investigate the associated anti-cancer mechanism. The results showed that FGBR inhibited preneoplastic lesions (ACF and SIM-ACF), reduced pro-inflammatory cytokines (TNF-α, IL-6 and IL-1β) and regulated apoptosis-related proteins (Bax, Bcl-2 and caspase-3). These findings suggest the potential of this novel fermented rice product for CRC chemoprevention.

Colonic ACF have been recognized as preneoplastic lesions of CRC. According to the fission mechanism, ACF are divided into proliferating ACF containing bifurcating crypts and quiescent/senescent ACF consisting of single crypts [27]. Proliferating ACF are found mostly in early colorectal carcinogenesis and may progress into larger ACF and tumors or may form quiescent/senescent ACF [27]. Quiescent/senescent ACF may enter the proliferation cycle again and then develop bifurcations or may disappear via apoptosis [27]. The present study demonstrated that all doses of FGBR (groups LF, MF and HF) significantly reduced the number of ACF with 1 AC, the primary ACF, suggesting that FGBR may inhibit the development of quiescent/senescent ACF by inducing their apoptosis or reversing them into normal crypts. GBR alone (group G) also showed a similar inhibitory effect on ACF. FGBR at the 2.5% level (group LF) further reduced the numbers of ACF with 2 and 3 ACs as well as total ACF and total AC, suggesting that it may suppress both proliferating and quiescent/senescent ACF.

Mucin production on crypts is another method for ACF classification. Dysplastic ACF can be distinguished from hyperplastic ACF based on mucin secretion [24,26]. Normal colonic crypts and hyperplastic ACF produce SUM, while dysplastic ACF produce either a mixture of SUM and SIM or only SIM [26,28]. An increase in SIM and a decrease in SUM secretion have been found during colorectal carcinogenesis. In CRC patients, the mucin change is presented by more SIM secretion and less SUM production [29]. In rats, normal colonic crypts and hyperplastic ACF show SUM staining, while dysplastic ACF show SIM staining, and the most dysplastic MDF do not show any mucin staining [30]. Therefore, the malignant degrees of preneoplastic lesions are in an increasing order of SUM-ACF, MIX-ACF, SIM-ACF and MDF. The present study showed that FGBR at the 2.5% level (group LF) significantly reduced the number of SIM-ACF and there was no MDF in this group. Rats fed FGBR at the 5% and 10% levels (groups MF and HF) had significantly higher numbers of MIX-ACF and slightly lower numbers of SIM-ACF compared with group D, showing a protection against the progression of MIX-ACF into SIM-ACF These results suggest that FGBR may regulate mucin alteration and low dose (2.5%) of FGBR may inhibit dysplastic ACF formation.

Pro-inflammatory cytokines have been shown to promote the development of sporadic CRC and colitis-associated cancer (CAC) [31]. The present study showed that both GBR (group G) and FGBR (groups LF, MF and HF) significantly decreased the secretion of TNF-α, IL-6 and IL-1β. These results suggest that GBR alone acts as a potent anti-inflammatory agent and that GBR fermented by *L. acidophilus* maintains this anti-inflammatory effect. A previous study demonstrated that GBR inhibited the formation of ACF and the expressions of β-catenin and cyclooxygenase-2 (COX-2) in azoxymethane (AOM)-induced rats [32]. Another study showed that the ethanol extract of germinated rough rice (GRR) displayed high hyperplastic ACF rather than dysplastic ACF and low β-catenin expression in AOM-treated rats [33]. GBR also had an anti-cancer activity in cell models. The bioaccessible fraction from parboiled GBR suppressed IL-8, monocyte chemoattractant protein-1 (MCP-1) and reactive oxygen species (ROS) in Caco-2 cells activated by H_2_O_2_ and IL-1β [34]. These studies indicated the importance of inflammatory factors in colorectal carcinogenesis and the potential of germinated rice products in suppression of CRC.

Several lines of evidence have demonstrated an anti-inflammatory activity of probiotics. For example, *L. acidophilus* decreased colonic leukotriene B4 (LTB4) production, inducible nitric oxide synthase (iNOS) expression and myeloperoxidase (MPO) activity in 2,4,6-trinitrobenzene sulfonic acid (TNBS)-induced rats [35]. *L. acidophilus* inhibited colitis by inducing goblet cell differentiation, interfering with endoplasmic reticulum stress and suppressing NF-κB activation in DSS-induced mice [36]. *L. acidophilus* DDS-1 upregulated IL-10 and downregulated TNF-α and IL-8 levels in lipopolysaccharide (LPS)-stimulated HT-29 cells [37]. Our previous study found that *L. acidophilus* exerted a potent anti-inflammatory effect via modulating the toll-like receptor 2 (TLR2)-mediated NF-κB and MAPK signaling pathways in LPS- and TNF-α-stimulated HT-29 cells [38]. These studies suggest that certain probiotics may be a potential adjuvant for the treatment of inflammatory diseases, including inflammatory bowel disease (IBD) and CAC.

Some fermented rice bran (FRB) products have been reported for high activities of antioxidation, anti-inflammation and anti-cancer [39]. An aqueous extract suspension of rice bran was fermented by lactic acid bacteria (LAB) and yeast to produce a novel fermented product, which showed a protective effect in DSS-induced IBD model mice [40]. This protective effect might be attributed to the starters showing an antioxidant activity [40]. Another FRB product was prepared by dual fermentation using fungus and mixed LAB [41]. Dietary supplementation of this FRB product effectively alleviated DSS-induced colitis in mice [41]. FBRA, a rice product fermented by *A. oryzae*, suppressed DSS-induced acute colitis in rats [42]. FBRA also inhibited CAC induced by DSS in *Apc*^Min/+^ mice [16]. The decrease in mRNA expressions of COX-2 and iNOS and the suppression of cell proliferation in the colon may be involved in chemopreventive effect of FBRA. [16]. In addition, FBRA lowered tumor incidence, reduced infiltrated inflammatory cells and decreased the expression of inflammation-related genes in QR-32 cells-gelatin sponge-induced mice, a model of inflammation-related carcinogenesis [17]. The anti-inflammatory effect of FBRA was associated with master regulating factors (such as TNF-α), which controlled the expression of adhesion molecules and chemokines at the inflamed site [17]. These findings suggest that certain fermented rice products may suppress both carcinogenesis and inflammatory responses.

Fermented GBR also shows an anti-inflammatory potential. In a mouse model of DSS-induced colitis, ethanol and ethyl acetate extracts of PBR, a product of *P. linteus* grown on GBR, ameliorated the pathological characteristics of colitis and reduced the expressions of NF-κB, iNOS, COX-2 and MAPKs [43]. The above authors supposed that PBR extract mitigates acute colitis through NF-κB-dependent pathways [43]. In addition, PBR extract showed a dose-dependent inhibition on nitric oxide (NO) and prostaglandin E_2_ (PGE_2_), reduced the mRNA expression of iNOS and TNF-α and inhibited the protein expression of iNOS, NF-κB and COX-2 in LPS-stimulated RAW264.7 macrophages [43]. The ethanol extract of CBR, a product of *A. camphorata* grown on GBR, improved DSS-induced colitis in mice and this effect was attributed to the reduction in iNOS, COX-2, TNF-α and IL-6 [44]. The inhibitory activity of CBR extract on these inflammatory mediators may be due to the regulation of the NF-κB and MAPK signaling pathways, and flavonoids may play a role in anti-inflammation [44]. The present study showed that both GBR and FGBR exerted an anti-inflammatory effect in DMH/DSS-treated rats. However, only FGBR at the 2.5% level (group LF) inhibited the development of advanced preneoplastic lesions (SIM-ACF and MDF). These findings suggest that FGBR may suppress colorectal carcinogenesis via other pathways.

Defects in the apoptotic pathway play a key role in carcinogenesis [45]. Many new treatment strategies targeting apoptosis are feasible and may be used in the treatment of various cancers, including CRC [45]. DMH caused alterations in proteins involved in the p53-mediated apoptotic pathway, and thus the expressions of Bax, caspase-3 and caspase-9 were low in DMH-treated rats [46]. Some rice products have been reported to promote cancer cell apoptosis by regulating pro-apoptotic and anti-apoptotic proteins [47]. The present study showed that both GBR (group G) and FGBR (group LF) significantly upregulated cleaved caspase-3 and downregulated Bcl-2 expression in DMH/DSS-treated rats. FGBR (group LF) further upregulated Bax expression. Moreover, the apoptosis-regulating efficacy of GBR and FGBR is similar to their inhibitory efficacy on preneoplastic lesions in the colon. These findings suggest that both GBR and FGBR may suppress colonic carcinogenesis through an apoptotic pathway. The anti-cancer effect of germinated rice has been observed in previous studies. The ethanol extract of GRR showed an antiproliferative effect on HCT116 cells [48]. The anti-cancer effect of GRR extract was higher compared with raw rough rice extract and associated with the increased antioxidative activity by germination [49]. Similarly, the ethanol extract of GBR showed antiproliferative, antioxidative and immunological activities in HCT116 cells [50]. Another study indicated that the ethanol extract of GBR had high inhibitory activity against HT-29 cells and the enhanced antiproliferative and antioxidative effects may be due to the increased bioactive compounds after germination [51]. γ-Aminobutyric acid (GABA), tocopherol, phytic acid and polyphenols show an antiproliferative effect against several types of cancer cells and germination further increases these compounds [51]. In addition to synthesis, the elevation of these compounds is probably due to the breakdown of cell wall and the activation of dormant enzymes during germination, which increase nutrients and bioactive compounds in rice grains [51].

Some previous studies have demonstrated the anti-cancer effect of *L. acidophilus*. One study used a mouse model of segmental orthotopic colon cancer in which animals were implanted with CT-26 murine colon cancer cells and found that *L. acidophilus* suppressed tumor growth and induced apoptosis [52]. Another study indicated that both supernatants and extracts from *L. acidophilus* inhibited Caco-2 cells by decreasing cell proliferation, migration and invasion, as well as by increasing cell apoptosis [53]. These studies suggest that *L. acidophilus* exerts an anti-cancer effect during colorectal carcinogenesis and that enhancement of cell apoptosis may be a critical mechanism implicated in CRC suppression.

The fermented rice product FBRA suppressed colonic ACF and tumors and decreased the proliferation of colonic mucosal cells in rats induced by AOM [15]. The water extract of FBRA caused an elevation of Bax and a reduction of Bcl-2 and led to caspase-3 activation in HCT116 cells [18]. This extract exerted oxidative damage to cancer cells and resulted in apoptosis by activating the mitochondrial pathway [18]. The above authors suggested that FBRA extract may contain a variety of bioactive oligopeptides, which are toxic to cancer cells [18]. Black rice bran (BRB) has been fermented by *Rhizopus oryzae* and/or *Rhizopus* *oligosporus* to produce fermented BRB (FBRB) [54]. The ethanol and methanol extracts of FBRB had higher total polyphenol content (TPC) compared with nonfermented BRB [54]. In addition, the antioxidative and antiproliferative activities of FBRB extract were also higher than those of nonfermented BRB [54]. The antioxidative activity of BRB is mainly influenced by phenolic acids bound to cell wall polysaccharides, and thus the lignocellulolytic activity of fungi may elevate the level and activity of free phenolics [54]. These findings confirm that fermentation is a critical method for the enhancement of chemopreventive components in rice.

The ethyl acetate extract of PBR induced the apoptosis of HT-29 cells and this effect was associated with upregulated p21, downregulated cyclin D1 and Bcl-2, released cytochrome c and activated caspase-3, caspase-8 and caspase-9 [19]. Some constituents, including ergosterol peroxide, GABA and β-glucan, were significantly increased by PBR treatment compared with *P. linteus* treatment alone [19]. Atractylenolide I might contribute to the anti-cancer effect of PBR [55]. The ethyl acetate extract of PBR also inhibited proliferation and metastasis of CT-26 cells via blocking the MAPK and PI3K/Akt signaling pathways, NF-κB and β-catenin [56]. The ethanol extract of PBR has been shown to increase the cetuximab sensitivity of KRAS-mutated colon cancer *in vitro* and *in vivo* [20]. Combined PBR extract and cetuximab acted synergistically against KRAS-mutant colon cancer cells and this synergistic effect could result from β-glucan [20]. Similarly, the ethanol extract of CBR inhibited HT-29 cell proliferation through inducing G0/G1 phase arrest and apoptosis by targeting β-catenin signaling [21]. The inhibitory efficacy of this extract on the proliferation of HT-29 and CT-26 cells was more effective than the extract of ordinary *A. camphorata*, while the ethyl acetate fraction of CBR showed the strongest inhibitory activity [21]. The ethyl acetate fraction of CBR contained adenosine, which induces apoptosis in various cancer cells through diverse signaling pathways [21]. Our previous study found that GBR combined with *L. acidophilus* and/or *B. animalis* subsp. *lactis* inhibited preneoplastic lesions by enhancing antioxidative capacity and inducing apoptosis in DMH/DSS-induced rats [22]. This synbiotics may be a potential chemopreventive agent against CRC [22]. GBR and probiotics may promote cell apoptosis via the p53-mediated pathway during colorectal carcinogenesis and the combination of GBR and *L. acidophilus* seems to be the most effective synbiotic [22]. These studies suggest that apoptotic pathways play an important role in CRC prevention exerted by fermented rice products including FGBR used in the present study.

Only limited studies have investigated the bioactive components of fermented GBR products. In a previous study, GBR and black rice were fermented with *A. oryzae* and then *L. acidophilus* to obtain fermented rice juice and sludge [57]. The protein contents in the sludge of both fermented GBR and fermented black rice were decreased, because protein was digested with metabolism of microorganisms [57]. In contrast, the GABA contents in the sludge and juice of both rice samples were increased by fermentation [57]. Fermented GBR had more protein and GABA than fermented black rice [57]. Another fermented GBR product was germinated native black rice juice fermented by *L. casei* TISTR 390, in which the titratable acidity and viable cell count increased, while the pH and total soluble solid decreased during 72-h fermentation [58]. The GABA level of this product was twice as high as that of the control group [58]. In addition, the free radical scavenging capacity showed that the half maximal inhibitory concentration (IC_50_) of this fermented GBR product was significantly lower compared with the control [58]. These studies confirm that germination plus fermentation may efficiently increase the GABA level and bioactivity of rice.

GABA inhibited the proliferation of HCT116, SW480 and SW620 cells and suppressed the metastasis of SW480 and SW620 cells [59]. In addition, GABA suppressed cell cycle progression through regulating the G2/M phase in HCT116 cells and G1/S phase in SW480 and SW620 cells [59]. There is accumulated knowledge on GABA application for human health accompanying with a demand on natural GABA supply [60]. It has been well known that germination significantly increases the GABA content of rice [61], suggesting that germinated rice is a good source of natural GABA. In addition, GABA produced by microorganisms can also fulfill the demand [60]. Many traditional foods produced by microbial fermentation provide safe and economic GABA and have the potential to be developed as novel health-promoting products rich in GABA [60]. There have been many studies focused on fermentation-mediated GABA production using bacteria, fungi and yeast [62]. The major GABA-producing microorganisms are LAB, which inhibit food pathogens and act as probiotics in the gastrointestinal tract [60]. It is commercially useful to produce GABA using LAB, because LAB can be served as starters of functional fermented foods [62]. For example, there is an enhanced production of GABA using rice bran extracts fermented by *Lactobacillus* *sakei* B2-16 [62]. Both germination and fermentation are common methods for GABA production, and so GABA may play an important role in suppressing colon carcinogenesis in the present study.

Although the bioactive components of fermented GBR have not been well documented, there have been some studies analyzing the changes in bioactive components of rice bran during fermentation. LAB fermentation could enhance the production of certain bioactive compounds and the antioxidant activity of rice bran [63,64]. The bioactive metabolites include lipids, proteins, essential amino acids, vitamin B complex, phenolic acids, γ-oryzanol, phytic acid and inositol [65]. A previous study extracted and quantified phenolics from heat-stabilized defatted rice bran (HDRB) using fermentation technology and found that fermentation by *B. subtilis* subsp. *subtilis* caused a significant increase in phenolic concentration and free radical scavenging capacity [63]. The fermented HDRB extract also had higher phenolic acids and (-)-epicatechin compared with nonfermented extract [63]. Fermentation of rice bran with *Pediococcus acidilactici*, *P. pentoseous* and *Lactococcus lactis* increased the concentration of phenolic acids, organic acids, γ-oryzanol and α-tocopherol [64]. In addition, the phenolic contents and antioxidant activity of rice bran showed significant improvement upon fermentation with *Lactobacillus lactic* or *L. plantarum* and the antioxidant activity of FRB extracts was positively correlated to their TPC [65]. Rice bran fermented with fungi also enhances the production of bioactive components [66,67]. The above studies suggest that fermentation causes significant changes in the nutrients and bioactive compounds of rice. These findings can also explain, at least partly, the chemopreventive effects of FGBR in the present study.

Interestingly, our study showed that only FGBR at the 2.5% level exerted a significant protective effect against DMH/DSS-induced advanced preneoplastic lesions (SIM-ACF and MDF) and there was no dose response. It seems that FGBR at the 10% level has no added benefit. This result may be related to both fermentation material and starter. It has been well known that rice contain various bioactive components with chemopreventive potential. Several methods have been used to enhance the concentrations of bioactive compounds in rice, such as germination and microbial fermentation [64]. However, previous studies showed inconsistent results of fermented rice products on cancer cells [68]. It may be due to the rice used, the difference in microbial metabolites and the influence of probiotics altering the bioavailability of anti-cancer compounds [68]. Plant phenolic compounds are often found in a biologically unavailable bound form due to an ester-bond to cell wall polysaccharides [68]. For example, phenolic compounds are present in rice bran at high levels; however, most of them are bound to arabinoxylans in the cell wall of bran [67]. Therefore, the optimal dose of rice products required to achieve anti-cancer concentrations of phenolic acids is unknown [68]. Generally, phytochemicals occur as glycoconjugates that exhibit lower bioavailability and bioactivity than their aglycone derivatives, which are smaller and less polar [69]. *L. acidophilus* metabolizes dietary plant glucosides into their bioactive aglycones, which are then exported from probiotics for absorption by the host and elicit various biological responses, most of which are beneficial [69]. However, the interfering effect on anti-cancer compounds induced by some free-form phytochemicals cannot be excluded. Fermentation is able to increase TPC because of the bioconversion of phenolic compounds from their conjugated forms to free forms [70]. During fermentation, the structural breakdown of grain cell walls results in higher bioaccessibility and bioavailability of bound and conjugated phenolic compounds [70]. Tannins released from their condensed forms upon fermentation may bind minerals such as calcium, and thus may reduce their bioavailability and bioactivities [70]. On the other hand, the released phenolic compounds may have an inhibitory effect on LAB during lactic fermentation [71]. The most abundant organic acids detected in fermented rice samples are lactic acid and acetic acid, which show significant elevation after LAB fermentation and lower the pH typically below 5 [11,64]. The acidic environment may be an adverse condition for the growth and function of some LAB [71].

During fermentation, the conversion of proteins and their digested products into ammonia, idol, phenols and biogenic amines by some bacteria may occur [72]. Biogenic amines, such as histamine and tyramine, are of concern, as they may be produced in high concentrations by microorganisms [72]. The intake of high levels of biogenic amines can induce allergic reactions [11]. Polyamines (spermine, spermidine and putrescine) have been involved in colorectal carcinogenesis [73]. Some structural components of probiotics are also bioactive and participate in the regulation of carcinogenesis. For example, the surface layer protein (SLP) from *L. acidophilus* protected HT-29 cells against intestinal pathogen-induced apoptosis through a mitochondria-mediated pathway [74]. The SLP inhibited the reduction of mitochondrial membrane potential, the increase of Ca^2+^ level and the activation of caspase-9 and caspase-3 in HT-29 cells [74]. Another study indicated that SLP from *L. acidophilus* did not induce apoptotic death in HCT116 cells [75]. Although the models are different, the anti-apoptosis activity of SLP produced by *L. acidophilus* may be one possible mechanism for the dose-independent effect of FGBR on colorectal carcinogenesis in the present study. FGBR at the 2.5% level may be an optimal dose for the chemoprevention of CRC, while FGBR at the 10% level seems to have no added benefit. The limitation of the present study is that only certain apoptosis-related proteins have been analyzed and they offer limited information regarding the characterization of possible mechanisms exerted by FGBR. Fermentation is a complex dynamic process associated with various biochemical reactions, and thus more studies are needed to elucidate the bioactive components of fermented rice products and their acting mechanisms.

## 5. Conclusions

In conclusion, the present study shows that FGBR, the germinated brown rice fermented by *L. acidophilus*, inhibits preneoplastic lesions of the colon in rats treated with DMH/DSS. Activation of the apoptotic pathway is a possible mechanism for this effect. FGBR at the 2.5% level may be an optimal dose for its preventive effect on colorectal carcinogenesis, while FGBR at the 10% level seems to have no added benefit. This fermented rice product may have the potential to be developed as a novel dietary supplement for the chemoprevention of CRC.

## Figures and Tables

**Table 1:** Effects of GBR and FGBR on DMH/DSS-induced ACF in the colon of F344 rats ^1,2^.

Group ^3^	ACF with			Number of ACF (number/cm^2^)	Number of AC (number/cm^2^)
	1 crypt	2 crypts	3 crypts		
D	2.2 ± 0.8 ^a^	3.3 ± 0.9 ^a^	1.5 ± 0.4 ^a^	7.8 ± 2.1 ^a^	16.6 ± 4.6 ^a^
G	1.6 ± 0.8 ^b^	2.8 ± 1.2 ^ab^	1.5 ± 0.8 ^a^	6.7 ± 2.7 ^ab^	14.8 ± 6.1 ^a^
LF	1.3 ± 0.4 ^b^	2.0 ± 0.7 ^b^	1.0 ± 0.3 ^b^	4.6 ± 0.9 ^b^	9.7 ± 2.0 ^b^
MF	1.5 ± 0.8 ^b^	2.7 ± 1.0 ^ab^	1.3 ± 0.7 ^ab^	6.1 ± 2.3 ^ab^	13.3 ± 5.2 ^ab^
HF	1.4 ± 0.6 ^b^	2.8 ± 1.5 ^ab^	1.4 ± 0.6 ^ab^	6.5 ± 2.8 ^ab^	14.7 ± 6.6 ^a^

^1^ All values are mean ± SD (n = 9–12). ^2^ Values with the same letter in a column are not significantly different from one another as determined by Duncan’s multiple range test, *p* < 0.05. ^3^ All rats were administered with DMH/DSS. D: AIN-93G diet, control; G: AIN-93G containing 10% GBR; LF: AIN-93G containing low dose (2.5%) of FGBR; MF: AIN-93G containing medium dose (5%) of FGBR; HF: AIN-93G containing high dose (10%) of FGBR.

**Table 2:** Effects of GBR and FGBR on the numbers of DMH/DSS-induced ACF according to the type of mucin and MDF in the distal colon of F344 rats ^1,2^.

Group ^3^	Number of ACF-Producing ^4^ (number/cm^2^)			MDF
	SUM	MIX	SIM	
D	8.5 ± 3.8 ^a^	0.9 ± 0.5 ^b^	2.0 ± 1.8 ^a^	0.12 ± 0.25 ^a^
G	8.2 ± 5.2 ^a^	1.4 ± 0.8 ^ab^	1.9 ± 2.1 ^a^	0.12 ± 0.22 ^a^
LF	6.1 ± 1.7 ^a^	1.1 ± 0.7 ^b^	0.3 ± 0.3 ^b^	0 ^a^
MF	8.4 ± 4.3 ^a^	2.1 ± 1.2 ^a^	1.0 ± 1.3 ^ab^	0.08 ± 0.26 ^a^
HF	9.2 ± 4.5 ^a^	2.3 ± 1.7 ^a^	0.9 ± 0.8 ^ab^	0.14 ± 0.22 ^a^

^1^ All values are the mean ± SD (*n* = 9–12). ^2^ Values with the same letter in a column are not significantly different from one another as determined by Duncan’s multiple range test, *p* < 0.05. ^3^ All rats were administered with DMH/DSS. D: AIN-93G diet, control; G: AIN-93G containing 10% GBR; LF: AIN- 93G containing a low dose (2.5%) of FGBR; MF: AIN-93G containing a medium dose (5%) of FGBR; HF: AIN-93G containing a high dose (10%) of FGBR. ^4^ SUM: sulfomucin; MIX: mixed sulfomucin and sialomucin; SIM: sialomucin; MDF: mucin-depleted foci.
